# The Inflation Reduction Act’s Impact Upon Early-Stage Venture Capital Investments

**DOI:** 10.1007/s43441-025-00773-3

**Published:** 2025-04-13

**Authors:** Duane G. Schulthess, Gwen O’Loughlin, Madeline Askeland, Daniel Gassull, Harry P. Bowen

**Affiliations:** 1 Vital Transformation LLC, 601 Pennsylvania Ave NW, South Building Suite 900, Washington, D. C., 20004 USA; 2 Vital Transformation LLC, Washington, D. C., USA; 3 McColl School of Business, Queens University of Charlotte, Charlotte, NC USA

**Keywords:** Inflation Reduction Act, IRA, Medicare, Small molecule, Large molecule, Venture capital

## Abstract

**Background:**

The Congressional Budget Office has stated there is no evidence of a systematic decrease in the percentage of venture capital flowing to pharmaceutical companies since IRA’s passage. This was echoed in Prof. Rita Conti’s September 17, 2024, Senate Finance Committee testimony.

**Methods:**

To test the IRA’s impacts on early-stage investments targeting therapeutics for the Medicare-aged population, a longitudinal dataset of commercially sponsored clinical trials by companies with a market valuation  ≤ $2 billion was obtained from the BioMedTracker database. These trials were filtered and curated to match early-stage investments to lead assets undergoing clinical development from January 1, 2018, to August 16, 2024.

**Results:**

From 161 lead assets with 619 individual investments, we find the aggregated total into large molecules in 2024 was 10 times larger than that for small molecules, which underwent a 68% decline after passage of the IRA. Individual investments made into small molecules decline by minus one-half as exposure to the Medicare-aged population increases after the passage of the IRA (p ≤ 0.0018). Testing large molecule investments by their exposure to Medicare post IRA’s passage is statistically inconclusive.

Research Conclusions.

This study presents evidence of a decline in the development of new therapies targeting the Medicare-aged population since the passage of the IRA. If these impacts were due to the economic downturn post-pandemic, we would observe statistically similar results in both large and small molecules. However, the results by molecule type diverge. Investors perceive large molecules to be of a lower investment risk relative to small molecules after IRA’s passage.

## Introduction

Introduced as The Build Back Better Act on September 27, 2021, the Inflation Reduction Act (IRA) was signed into law by the Biden Administration on August 16, 2022, for the first time, “allowing Medicare to negotiate prescription drug costs.” [1].

The Congressional Budget Office (CBO), in their scoring of the IRA, reported that it would reduce direct spending on drugs by $249 billion. CBO further stated that the impact would be limited as “the number of drugs that would be introduced to the U.S. market would be reduced by about two over the 2023–2032 period, about five over the subsequent decade, and about eight over the decade after that.” [2].

Many leading academics have echoed this belief that the IRA is relatively benign for the U.S. biopharmaceutical innovation ecosystem. In a September 17, 2024, Senate Finance Committee hearing, [3] Prof. Rena M. Conti presented research stating they “found little evidence the IRA has resulted in a meaningful decrease in the level of venture capital (VC) investment in new drug development.” [4]. Prof. Conti also said that “Late-stage private company and public equity valuations, IPOs, follow-on offerings, and mergers and acquisitions in biopharma largely held steady in the 18 months after passage of the IRA and [that such investments] have had a positive start in 2024.” [5].

The IRA allows the Center for Medicare and Medicaid Services (CMS) to set prices for the top 20 drugs by spending beginning year 9 for small molecules and year 13 for large molecules, creating a two-tiered reimbursement system. The challenge for investors then, particularly those operating at the early stages of drug development, is that the IRA’s provisions create specifically divergent financial disincentives for large and small molecule therapies, including market risks as measured by a disease’s prevalence in the Medicare-aged population.

The research cited above suggests the IRA poses little risk to biopharmaceutical development. However, the methodological approach taken in these studies only examines the IRA’s impacts in the aggregate, i.e., only the average impacts. They do not segment by large or small molecules, measure a specific indication’s exposure to the Medicare-aged population, or differentiate late-stage phase III research from earlier stages in smaller, highly innovative firms where the IRA’s impacts are more likely to first appear due to shifts in VC or angel investing behavior.

There is evidence that VCs and developers are responding to the IRA’s disincentives. For example, on February 9, 2024, Suneet Varma, commercial president of Pfizer Oncology, stated, “Biologics [large molecules represent] a more durable revenue potential based on several factors, including differentiated access and affordability to the patient, IRA considerations and patent expiration timeline.” [6] Similarly, venture capitalist Peter Kolchinsky stated, “We’ve told our companies... stay away from any disease of aging where you’re going to be heavily dependent on Medicare.” [7].

In addition to potentially missing these changes in investor behavior, the aggregated approach of prior research is also likely to underestimate the IRA’s microeconomic impacts by not capturing the relationship between Biopharmaceutical profits and research & development (R&D). Specifically, as recently highlighted by Chandra et al., “The top 20 companies by revenue (accounting for 71% of total revenue) contribute 50% of R&D investment.” [8]. This implies that the Biopharmaceutical sector's profits and R&D spending follow the Pareto principle, whereby 20% of successful companies fund an outsize portion of the entire R&D ecosystem [9]. As the total R&D ecosystem will see revenues and profits substantially reduced due to the IRA restricting to 9 years the sales of small molecule therapies treating the Medicare aged population, we would expect the first responses to the law’s disincentives to be observed among therapies with a small molecule modality.

Early-stage investors will anticipate a drop in the future value and profitability of companies exposed to the 9-year mandatory price setting clauses of the IRA for small molecules when compared to the 13 years of sales allowed for large molecules. This should lead to an observable shift in investor behavior before and after IRA’s introduction as well as between small and large molecules, and a decline in the relative value of small molecules with a high exposure to the Medicare aged population.

Vogel et al. said, “Capital already committed to biopharmaceutical VC funds will be deployed even if return expectations have shifted. This is not necessarily true for VC funds seeking new capital.” [10]. Consistent with this remark, this paper uses both descriptive statistics and formal statistical tests to detect changes in investing behavior concerning early-stage funding of large and small molecules and in developing assets with high exposure to the Medicare-aged population and therefore IRA price setting. The analysis is conducted using a purposely constructed dataset of recent investments made by early-stage VC and angel investors, segmented at the indication and molecule level.

## Materials and Methods

Our dataset was built by extracting data from BioMedTracker [11] and ClinicalTrials.gov for the period January 1, 2018 to May 6, 2024 on U.S. companies with a market capitalization/valuation of less than or equal to $2 billion, which is the standard forward-looking risk-weighted net present value of a VC’s successful exit at the time of a therapeutic product’s FDA submission for marketing authorization [12]. The $2 billion cutoff was chosen to ensure our cohort would focus on small to mid-sized firms and include drugs under clinical development requiring early-stage funding for therapies that would potentially be subjected to IRA negotiations if eventually commercialized. This cohort comprised 1,137 clinical trials.

As many molecules undergoing clinical trials will be developed for multiple indications simultaneously, and drugs often change names or are redeveloped under altered formulations, each of the 1,137 individual clinical trials was researched on a company-by-company level to determine which program was functioning as a given company’s “lead” asset. To identify a company’s lead asset, all clinical trials for which the FDA disclosed the company as the lead sponsor were searched to determine which asset was at the highest phase of development, with a starting date on or before January 1, 2018 through the date our data was compiled on August 16, 2024.

As our objective in this analysis is the detection of any meaningful change in investor behavior between large and small molecules before and after the introduction of the IRA, our selection of a lead asset was not dependent upon if the trial was discontinued or operational at the time of our analysis. The lead asset was categorized by the date when the trial was launched according to the start date listed in the FDA’s clinicaltrails.gov database (see Data and Statistical Appendix).

In several cases, our lead asset selection process found that a molecule was being developed for either an accelerated approval pathway, an orphan pathway, or both simultaneously and with varying indications. In these cases, the indication with the largest potential market size was selected as the company’s leading asset.

There were also several cases of an investigational therapy that had undergone multiple failed phase II clinical trials and had then been registered in a clinical trial addressing the COVID-19 pandemic. We regarded these trials as merely opportunistic and opted to use the earlier, non-COVID-19 indication as the lead asset, except in those rare cases where investors had shown significant financial interest in the COVID-19 trial where it was legitimately a company’s lead asset.

Once lead assets by company were identified, a forensic audit of all identifiable individual investments into those companies by the date they were made between January 1, 2018, and August 16, 2024 was executed using the combined resources of Pitchbook, BioMedTracker, SEC filings, press releases, and published annual corporate reports. These individual investments were then categorized and linked by the date they were made to the company’s lead asset by the start date of the corresponding clinical trial phase.

This process yielded 228 lead assets, with 897 individual investments. These lead assets were then segmented by indication and clinical trial phase to focus on only those lead assets in either phase I or II clinical trials, yielding 161 early-stage lead assets with 619 total individual investments, 327 into large molecules, and 292 for small molecules. (Table 1).

**Table 1:** showing the total number of investments made into lead assets by modality undergoing phase I and II clinical trials with a start date between January 1, 2018, and August 16, 2024, for companies ≤ $2 billion valuation

	Lead Assets	Phase 1 Investments	Phase 2 Investments	Total Investments
Biologic (Large Molecule)	78	71	256	327
New Molecular Entity (Small Molecule)	83	74	218	292
Total	161	145	474	

Our investment criteria focused on capital raised from VC, angel, equity, partnering, initial public offerings (IPO), acquisitions, and licensing activity. Debt assumed by the developing company is excluded. Where a clear audit trail of investment or ownership was not possible, those companies and developments were excluded from our analysis.

Our analysis focuses on Type-1 novel FDA-approved therapies. Follow-on indications or post-approved combination therapies are excluded. The investment data are measured in July 2024 constant dollars. In the preparation of the data for this research, a statistical test for a difference in the median size of phase 1 versus phase 2 investments was run. We found no statistical difference in their median values (p ≤ 0.3432) and thus treat both phases of investments equally.

To determine a lead asset’s exposure to the Medicare Aged population as a variable for investor risk, 133 separate indications drawn from the FDA’s clinical trial data registry for our 161 lead assets were researched by disease prevalence, to determine the percentage of the population for that indication over 65 years of age. This prevalence data by age and indication was drawn from peer reviewed sources in the common body of knowledge (see Data and Statistical Appendix).

Table 2 provides information on the specific statistical tests, datasets, dependent variables, and independent variables used in our research to determine the impact of IRA upon early-stage investors and their willingness to invest in therapies targeting the Medicare-aged population.

**Table 2:** Statistical operations, dates of data, dependent and independent variables in our paper with corresponding figures and tables highlighting results

ID	Description	Date range	Pop (N)	Test Type	Dependent Variable	Independent Variable(s)
Figure 1	Test of all Phase 1 & 2 clinical trial starts before and after IRA's introduction	1/1/2018—12/31/2023	1,100	Linear regression	Monthly clinical trial starts	IRA's Introduction on 9/27/21 as a categorical variable (1 or 0)
Figure 3	Test of investments made into Phase 1 & 2 small molecule lead assets before and after IRA's introduction	1/1/2018—8/16/2024	292	Kruskal–Wallis test at median	Median size of investments into small molecules	IRA's Introduction on 9/27/21 as a categorical variable (1 or 0)
Figure 4	Test of investments made into Phase 1 & 2 large molecule lead assets before and after IRA's introduction	1/1/2018—8/16/2024	327	Kruskal–Wallis test at median	Median size of investments into large molecules	IRA's Introduction on 9/27/21 as a categorical variable (1 or 0)
Table 3	Test of investments made into lead assets with a high Medicare exposure classified by indication, before and after IRA's introduction	1/1/2018—8/16/2024	244	Kruskal–Wallis test at median	Median size of investments	IRA's Introduction on 9/27/21 as a categorical variable (1 or 0), Exposure ≥ 60% of median indication prevalence for those over 65 years of age
Figure 5 Small Above Median	Test for change in median size of investments into small molecules after IRA’s introduction—high Medicare exposure	1/1/2018 – 8/16/2024	99	Multiple linear regression	Natural log (ln) of small molecule investments	IRA's Introduction on 9/27/21 as a categorical variable (1 or 0), Exposure ≥ 60% of median indication prevalence for those over 65 years of age
Figure 5 Small Below Median	Test for change in median size of investments into small molecules after IRA’s introduction—low Medicare exposure	1/1/2018 – 8/16/2024	193	Multiple linear regression	Natural log (ln) of small molecule investments	IRA's Introduction as a categorical variable (1 or 0), Exposure ≤ 59% of median indication prevalence for those over 65 years of age
Figure 5 Large Above Median	Test for change in median size of investments into large molecules after IRA’s introduction—high Medicare exposure	1/1/2018 – 8/16/2024	153	Kruskal–Wallis test at median	Median size of investments into large molecules	IRA's Introduction on 9/27/21 as a categorical variable (1 or 0), Exposure ≥ 60% of median indication prevalence for those over 65 years of age
Figure 5 Large Below Median	Test for change in median size of investments into large molecules after IRA’s introduction—low Medicare exposure	1/1/2018 – 8/16/2024	174	Kruskal–Wallis test at median	Median size of investments into large molecules	IRA's Introduction on 9/27/21 as a categorical variable (1 or 0), Exposure ≤ 59% of median indication prevalence for those over 65 years of age
Figure 5 Large and Small Above Median	Test comparing change in median size of investments into large and small molecules with high Medicare exposure after IRA’s introduction	1/1/2018 – 8/16/2024	99 (s), 153 (lg)	Multiple linear regression	Natural log (ln) of small and large molecule investments	IRA's Introduction on 9/27/21 as a categorical variable (1 or 0), Exposure ≥ 60% of median indication prevalence for those over 65 years of age
Figure 6	Model testing the change investment size as Medicare exposure increases above median	1/1/2018 – 8/16/2024	77 (s), 117 (lg)	Multiple linear regression	Natural log (ln) of small and large molecule investments	IRA's Introduction on 9/27/21 as a categorical variable (1 or 0), Exposure ≥ 63% of median indication prevalence for those over 65 years of age

## Results

### Drug Development Impacts

**Figure 1:**
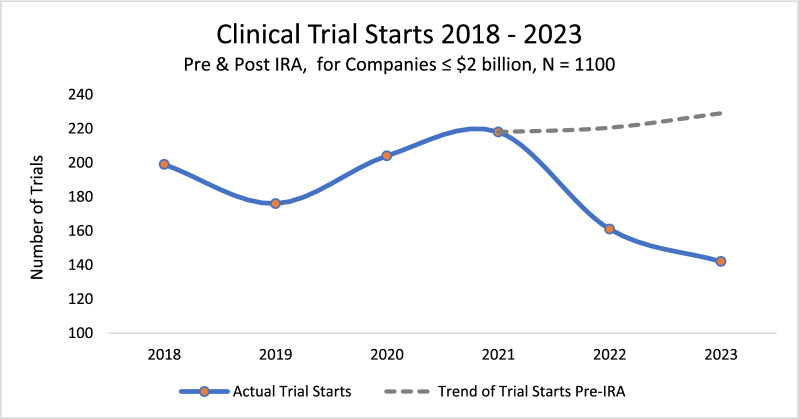
Number of clinical trials launched between 2018 and 2023 shows a statistically significant (p ≤ 0.0179) decline when calculated at the mean. Graph shows a 35% decline after the introduction of the IRA on September 27th, 2021. Data cohort is 1,100 phase I and II clinical trials extracted from BioMedTracker for companies ≤ $2 billion valuation.

Figure 1 shows the evolution in all clinical trial launches by our cohort of companies between 2018 and 2023, which reveals a 35% decline in clinical trial starts after the introduction of the IRA on September 27, 2021. A regression test for the difference in the mean value of monthly clinical trial starts in the years before and after the introduction of the IRA indicates a statistically significant difference (p ≤ 0.0179). This result was also duplicated by recently published research by the National Pharmaceutical Council [13].

### Impacts on Investor Behavior – Size of Investments

To detect any change in investor behavior by small or large molecule modality due to the introduction of the IRA, the following analysis focuses on our data that links all investments made between January 1, 2018, through August 16, 2024, specifically to the development of lead assets.

**Figure 2:**
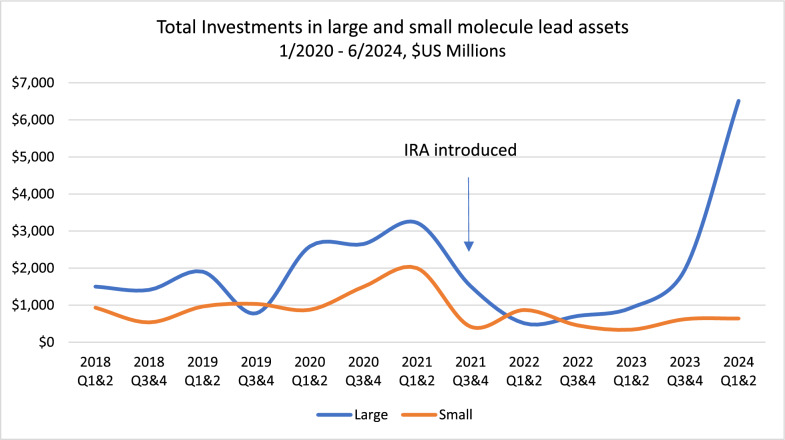
Graph showing shift in early-stage (phase I and II) investments into small and large molecule lead assets. Data for U.S. companies with a market value ≤ $2 billion, values in 2024 constant dollars, 1/1/2018 – 6/30/2024.

Figure 2 shows total investments into our cohort of 161 lead assets from January 1, 2018, through June 30, 2024, segmented by large and small molecules. As evident in Fig. 2, aggregate investments in large molecule assets underwent a significant decline starting in 2021, coinciding with the passage of the IRA, but thereafter rose substantially, with aggregate large molecule investments being 10 times larger at $6.5 billion than those of small molecules totaling $640 million as of June of 2024.

Conversely, Fig. 2 shows that aggregate investments in small molecules declined 68% since their peak before the introduction of the IRA, from $2 billion to $640 million, also demonstrating a marked decrease in total lead asset investments into small versus large molecules.

**Figure 3:**
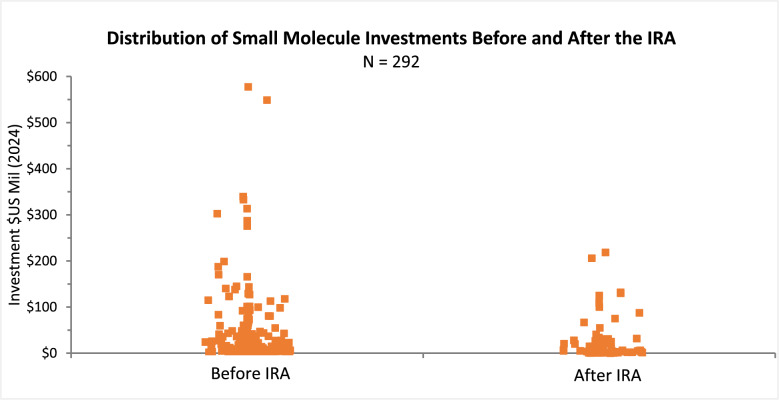
Dot plots show distributions, before and after IRA’s introduction, of the size of early stage (phase I and II) investments into small molecule lead assets. Data are for 1/1/2018 – 8/16/2024 on U.S. companies with a market value ≤ $2 billion, investment values in 2024 constant dollars.

Figure 3 provides further evidence of the decline in small molecule investments by showing the distribution of the size of investments into small molecules for the periods before and after the introduction of the IRA. Based on these data, a Kruskal–Wallis test indicates a statistically significant difference (p ≤ 0.0099) in the median size of small molecule investments, affirming their decline after IRA’s introduction observed in Fig. 2.

**Figure 4:**
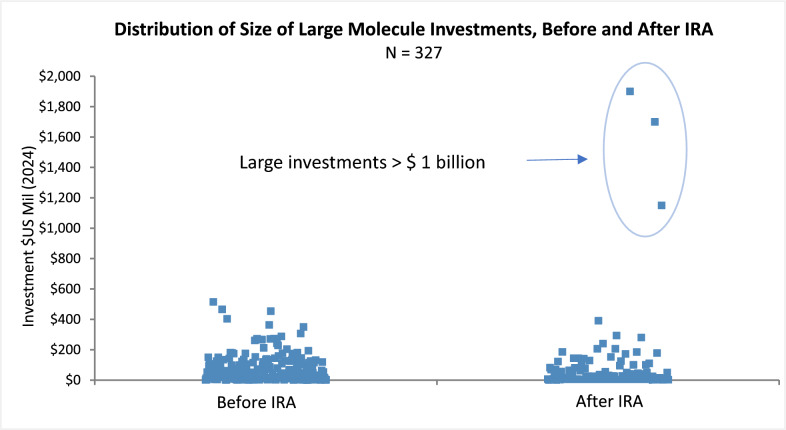
Dot plots show distributions before and after the introduction of the IRA on the size of early stage (phase I and II) investments into 327 large molecule lead assets. Data are for 1/1/2018 – 8/16/2024 on U.S. companies with a market value  ≤ $2 billion, investment values in 2024 constant dollars. Investment outliers are circled.

For large molecules, the Kruskal–Wallis test indicates a statistically significant decline (p ≤ 0.0013) in the median size of large molecule investments before and after the introduction of IRA (Fig. 4). The previously observed increase in total investments in large molecules (Fig. 2) is due to three large post-IRA investments, each over $1 billion. The Medicare exposure for IRA price setting for these investment outliers is near or below our median of 59% as calculated by the indication prevalence for those over 65 years of age in our cohort. They are HER2 + Breast Cancer with 50% exposure, alpha-1 antitrypsin deficiency (dAATD) with 27% exposure, and kidney transplant rejection with 60% exposure [14].

### Impacts on Investor Behavior – Exposure to the Medicare-aged Population

To further assess the evolution of early-stage investor behavior regarding lead assets, we examined investments in relation to their exposure to the Medicare-aged population. Specifically, we filtered our 161 lead assets to select only those indications whose exposure to the population over 65 years of age exceeded the median indication prevalence value of 59% across our cohort. Within this sub-sample, the median size of the 244 individual investments into these lead assets shows a statistically significant (p ≤ 0.008) decline of 51% after IRA’s introduction (see Data and Statistical Appendix). Aggregated over indications, the second to last row of Table 3 indicates a 74% decline ($175 mil to $45 mil) in the median size of investments after IRA’s introduction.

**Table 3:** Indications in our cohort above the median of 59% of Medicare exposure for those over 65 years of age as calculated by indication prevalence for U.S

Indication	Number of Investments	Prevalence in Medicare-Aged Population	Investments ($ mil.)	
			Before IRA	After IRA
Non-Small Cell Lung Cancer (NSCLC)	36	72%	$2,045	$1,040
Alzheimer's Disease (AD)	30	95%	$178	$108
Multiple Myeloma (MM)	26	65%	$1,936	$843
Acute Myelogenous Leukemia (AML)	25	61%	$1,036	$535
Pancreatic Cancer	13	69%	$267	$302
Head and Neck Cancer	11	70%	$123	$59
Prostate Cancer	11	63%	$1,721	$43
Gastric Cancer	10	60%	$337	$72
Non-Tuberculous Mycobacteria (NTM)	8	60%	$178	$85
Wet Age-Related Macular Degeneration (Wet AMD)	8	90%	$200	$626
Acute Renal Failure (ARF)	7	67%	$176	$0
Cartilage and Joint Repair	7	75%	$49	$14
Amyotrophic Lateral Sclerosis (ALS)	6	64%	$68	$204
Idiopathic Pulmonary Fibrosis (IPF)	6	90%	$110	$37
Kidney Transplant Rejection	6	60%	$324	$1,347
Rheumatoid Arthritis (RA)	6	61%	$44	$0
Acute Decompensated Heart Failure (Acute HFrEF)	5	80%	$175	$16
Dementia	4	95%	$716	$0
Autoimmune Disorders	3	61%	$50	$0
Mesothelioma	3	80%	$380	$0
Renal Cell Cancer (RCC)	3	71%	$35	$47
Stroke Prevention in Atrial Fibrillation (SPAF)	3	76%	$0	$15
Bladder Cancer	2	75%	$51	$45
COVID-19 Treatment	2	65%	$24	$0
Cerebral Edema	1	80%	$2	$0
Chronic Cough	1	90%	$44	$0
Influenza	1	65%	$0	$132
Totals	*244*	*-*	*$10,268*	*$5,569*
Mean	*9.04*	*73%*	*$380*	*$206*
Median	***6***	***70%***	***$175***	***$45***
Std. Dev	*9.15*	*11%*	*$584*	*$351*

Companies ≤ $2 billion market value

Investments measured in 2024 constant dollars are for phase I and II from 1/1/2018 – 8/16/2024

Using the Kruskal–Wallis test, an observed difference (decline) of 51% in the median investment size by indication before and after IRA’s introduction is statistically significant (p ≤ 0.008)

Aggregated over all indications, the median size of investments declined 74% (from $175 mil. to $45 mil.)

**Figure 5:**
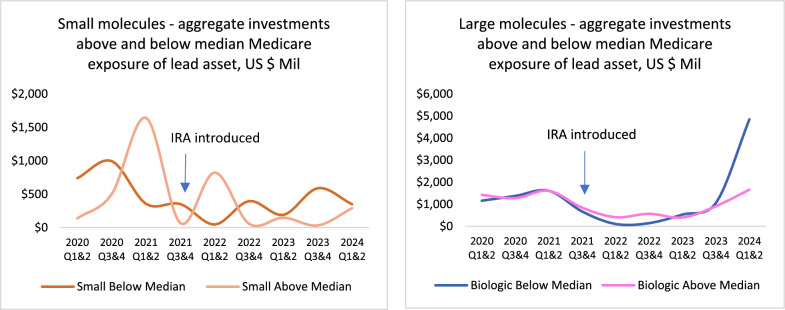
Shifts in early-stage (phase I and II) investments for small and large (Biologic) molecule lead assets above and below the median (59%) exposure by indication prevalence for those 65 years of age or older before and after introduction of the IRA. Data for U.S. companies with a market value ≤ $2 billion, values in 2024 constant dollars, 1/1/2020 – 6/30/2024.

By segmenting the size of investments into lead assets by high and low Medicare exposure before and after the introduction of the IRA, we can determine if the IRA impacted the decisions of early-stage investors by molecule type and risk of price setting. For small molecule investments, Fig. 5 shows that, after IRA’s introduction, there was a relative increase in investments that target indications below the median value (59%) of exposure to the Medicare-aged population.

Regressing the natural logarithm (ln) of the size of investments from 1/1/2018 through 8/16/2024, we find that the size of small molecule investments above the median exposure to the Medicare-aged population (n = 99) show a statistically significant decline after the introduction of the IRA (p ≤ 0.0488). Conversely, for small molecules below the median of exposure to the Medicare-aged population (n = 193) we find no significant change in the size of investments after the introduction of the IRA (p ≤ 0.1748).

Similar testing for large molecules showed the median size of investments by high (n = 153, p ≤ 0.023) or low (n = 174, p ≤ 0.0313) exposure to the Medicare-aged population declined before and after the IRA’s introduction – exposure for large molecules to price setting for indications targeting the Medicare aged population at year 13 due to the IRA did not alter the median size of investments.

When comparing the median size of investments into small and large molecules with a high exposure to the Medicare-aged population before and after IRA’s introduction, we find that the median size of investments for small molecules (n = 99) declined by roughly 7% more than the investments made into large molecules (n = 153). This decline in the median size of investments was further investigated for significance by regressing the natural log of investments in the sample of indications with above the median Medicare exposure on a dummy variable for before and after IRA, and for large and small molecules. The results indicated a greater rate of the decline in the investment size of small molecules compared to those into large molecules after IRA’s introduction (p ≤ 0.0002) (see Data and Statistical Appendix).

**Figure 6:**
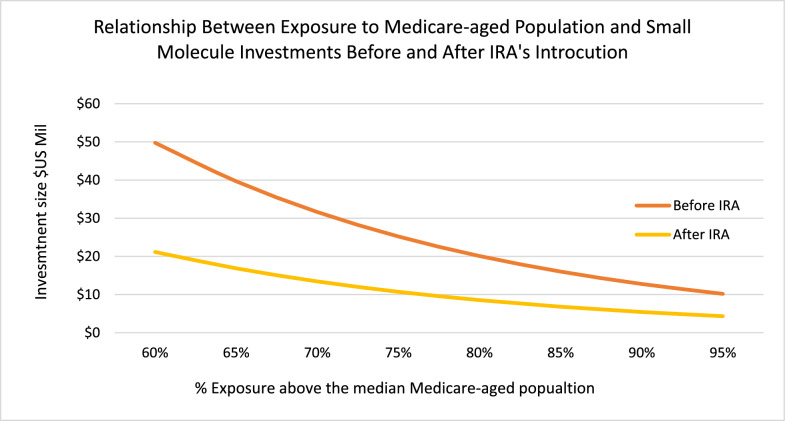
Estimated multiple regression model of the relationship between small molecule investments and exposure to Medicare-aged population above the median (≥ 63%) and time periods pre—and post the introduction of the IRA. Data for 1/1/2018 – 8/16/2024 on U.S. companies ≤ $2billion valuation. Investments for phase I and II measured in 2024 constant dollars at the natural log (n = 77).

To test the impact of increasing an indication’s exposure to the Medicare-aged population on the size of small molecule investments, we modeled the natural log of investments as a function of an indication’s exposure to the Medicare-aged population, and a dummy variable for investments made pre- and post-IRA’s introduction. The results of this model indicated a statistically significant negative relationship for investments into small molecules as exposure to the Medicare-aged population increases. Investments decline by 57% after IRA’s introduction as exposure to the Medicare population increases above the median of 59% (p ≤ 0.0018) (Fig. 6).

If the observed impact on small molecule investments is due to the general post-pandemic economic environment and not the IRA, as stated in the October 27, 2024, letter from Phillip L. Swagel, Director of the CBO [15], we would expect to observe similar results in large molecules. However, in multiple statistical tests of the median and the natural log of large molecule investments by an indication’s exposure to the Medicare-aged population and a dummy variable for investments made pre- and post-IRA, the results showed no statistically significant difference in the median of large molecule investments before and after the IRA's implementation.

In addition, the median frequency of an investment’s large molecule exposure to the Medicare-aged population is virtually unchanged before and after the introduction of the IRA at 59.6% and 59% respectively. We interpret these results to mean that, post-IRA, investors perceive large molecules to be a lower investment risk than small molecules.

## Discussion

The disconnect between IRA’s impacts upon early- and late-stage drug discovery has been discussed and debated, most notably between the CBO and the U.S. House of Representatives Budget Committee Health Care Task Force [16]. Many of the previous studies claiming negligible impacts of the IRA have incorporated institutional large-scale financing of late-stage phase III therapies and have not explicitly investigated smaller companies targeting novel therapeutics and mechanisms of action reliant upon early-stage venture capital financing, initial public offerings, angel investors, etc.

The analysis of this paper found that, at the cohort level, early-stage investors and small companies with assets under development consciously changed their behavior and investment activity in response to the altered financial risks caused by the introduction of the IRA. As the average length of phase II and III clinical trials is roughly 40 months each, we expect these reductions to impact the rate of FDA approvals in 5 to 6 years [17].

This analysis also found evidence of a decline after IRA’s introduction in the size of investments into small molecules based upon an indication’s exposure to the Medicare-aged population; small molecules targeting indications with a high exposure to the Medicare-aged population saw their investments decline in size. In contrast, small molecules with a low exposure to Medicare after IRA’s introduction had no statistical decline in investments. This suggests early-stage investors are now favoring large molecule indications with high exposure to the Medicare-aged population due to a change in the perceived risks of investing in small versus large molecule assets.

No change was observed in the size of investments into large molecules based on exposure to the Medicare-aged population. Large molecule investments were broadly similar after IRA’s introduction regardless of an indication’s likelihood of seeing ‘maximum fair pricing’ set in year 13 after a large molecule drug is approved. Further, relative to large molecules with a high exposure to Medicare, investments into small molecules with a high exposure to the Medicare aged population saw significantly greater declines in the size of their investments compared to similarly exposed large molecules.

Further support for the thesis of reduced investments in small molecule therapies with high exposure to the Medicare-aged population was the presence of three significant outlier investments into large molecules, each over $1 billion, undertaken after the passage of the IRA. This highlights the lower relative risk and potentially higher return to investments in large molecules compared to small molecules since, under the IRA, Medicare sets prices for large molecules in year 13 but in year 9 for small molecules, thereby enhancing revenue generation for large molecule relative to small molecule therapies.

## Limitations

According to Vogel et al., “In 2023, biopharmaceutical VC funds reportedly raised $21 billion in new capital, less than the $31 billion peak in 2021.” [18]. Our research does not dispute that a downturn in the biopharmaceutical market may be impacting our results. However, our study does demonstrate significant changes in investing behavior that are predictable based on hypothesized impacts of the IRA. Such changes are evident in the observed movements away from small molecule investments for indications above the median exposure to the Medicare-aged population, the increased size of aggregate investment into large molecules for indications with a median or below exposure to the Medicare-aged population, as well as the unchanged frequency of investments into large molecules when compared to small molecules relative to their exposure to the Medicare-aged population.

This paper uses the median age weighted exposure of 59% calculated by indication prevalence above and below 65 years of age as a proxy for an investors’ potential exposure and risk due to the introduction and passage of the IRA. While total U.S. population by indication prevalence was also calculated, when this independent variable was incorporated into our analysis, it was not statistically significant and weakened our regression output (p ≤ 0.7521). Exposure to the Medicare population by indication prevalence above and below 65 years of age was a more robust predictor of investor behavior than the potential size of the market in our cohort, but it is nonetheless a surrogate measure for investor risk associated with the IRA.

## Conclusion

Prior research on the potential impacts of the IRA on the biopharmaceutical ecosystem has primarily focused on the aggregate (mean) impacts and failed to examine segmented investments by indication or the degree of an indication’s exposure to the Medicare-aged population. As a result, prior research has obfuscated and primarily overlooked the IRA’s impacts on early-stage investment and drug development behavior. As the development time for new therapies is roughly 10 years from an FDA Investigational New Drug application to approval [19], the evidence presented in this paper of an observed 35% decline in early-stage clinical trial launches, a 74% drop in the median size of aggregate investments into indications specifically targeting the Medicare aged population, a statistically significant drop after IRA’s introduction in the median size of the investments made into small molecules versus those made into large molecules, and a 68% decline in funding for early-stage developments in small molecules, is evidence that the IRA has created negative impacts upon the very population the legislation is allegedly designed to aid. Namely, the Medicare-aged population who require effective new therapies in areas of high unmet medical need, but who are likely to have fewer new treatments available in the future.

## Data Availability

All data used in this analysis are available in an accompanying Data and Statistical Appendix. IRA VC Dataset (2).zip
